# The *Evx1/Evx1as* gene locus regulates anterior-posterior patterning during gastrulation

**DOI:** 10.1038/srep26657

**Published:** 2016-05-26

**Authors:** Charles C. Bell, Paulo P. Amaral, Anton Kalsbeek, Graham W. Magor, Kevin R. Gillinder, Pierre Tangermann, Lorena di Lisio, Seth W. Cheetham, Franziska Gruhl, Jessica Frith, Michael R. Tallack, Ke-Lin Ru, Joanna Crawford, John S. Mattick, Marcel E. Dinger, Andrew C. Perkins

**Affiliations:** 1 Mater Research, Translational Research Institute, University of Queensland, Brisbane, Queensland, 4101, Australia; 2 The Institute for Molecular Bioscience, University of Queensland, Brisbane, Queensland, Australia; 3 Garvan Institute of Medical Research, Sydney, Australia; 4 Diamantina Institute; Translational Research Institute, University of Queensland, Brisbane, Queensland, 4102, Australia; 5 The Australian Institute for Bioengineering and Nanotechnology, University of Queensland, Brisbane, Queensland, 4102, Australia; 6 St Vincents Clinical School, Faculty of Medicine, UNSW Australia, Sydney, Australia; 7 The Princess Alexandra Hospital, Brisbane, Queensland, 4102, Australia

## Abstract

Thousands of sense-antisense mRNA-lncRNA gene pairs occur in the mammalian genome. While there is usually little doubt about the function of the coding transcript, the function of the lncRNA partner is mostly untested. Here we examine the function of the homeotic *Evx1*-*Evx1as* gene locus. Expression is tightly co-regulated in posterior mesoderm of mouse embryos and in embryoid bodies. Expression of both genes is enhanced by BMP4 and WNT3A, and reduced by Activin. We generated a suite of deletions in the locus by CRISPR-Cas9 editing. We show EVX1 is a critical downstream effector of BMP4 and WNT3A with respect to patterning of posterior mesoderm. The lncRNA, *Evx1as* arises from alternative promoters and is difficult to fully abrogate by gene editing or siRNA approaches. Nevertheless, we were able to generate a large 2.6 kb deletion encompassing the shared promoter with *Evx1* and multiple additional exons of *Evx1as.* This led to an identical dorsal-ventral patterning defect to that generated by micro-deletion in the DNA-binding domain of EVX1. Thus, *Evx1as* has no function independent of EVX1, and is therefore unlikely to act *in trans*. We predict many antisense lncRNAs have no specific *trans* function, possibly only regulating the linked coding genes in *cis*.

Gastrulation is a critical early developmental process in which mesoderm and definitive endoderm are generated, and then specified. The three germ layers (ectoderm, mesoderm and definitive endoderm) eventually form all of the tissues of the adult organism. In the mouse, a proximal-distal (P-D) axis is established first from ~E5.0 in response to nodal signalling, then an anterior-posterior (A-P) axis is established during gastrulation in response to BMP, FGF and WNT morphogen gradients^1^,^2^. Specification of epiblast cells into mesendodermal cell fates occurs as they migrate through the primitive streak and activate transcriptional programs in response to these gradients^3^. The cells which migrate first are specified to a posterior fate in response to high local concentrations of BMPs, WNTs and FGFs. These cells form blood, the vasculature and heart. Cells which migrate last through the streak take on an anterior fate in response to high local concentrations of activin/nodal morphogens and inhibitors of BMPs and WNTs such as Chordin, Noggin, Dkk1, Cerberus-like 1 (Cer1) and Lefty 1 and 2^2^. They form notochord, somites and definitive endoderm. Cells from the mid-streak region form intermediate mesoderm derivatives such as kidney.

Hox genes have an established role in patterning during bilaterian embryonic development^4^. *Evx1* is located 50 kb downstream of the HoxA cluster^5^. Individual members of this cluster have graded anterior boundaries of expression and control rostral-caudal neural fates^4^. *Evx1* is expressed in the posterior primitive streak from ~E6.5^6^, which is earlier in development than any members of the HoxA cluster^7^. Therefore, it is considered not to be co-regulated with the HoxA cluster during gastrulation. In Xenopus and zebrafish, the homologs of EVX1 (*xhox3, eve1*) play roles in ‘posteriorization’ of nascent mesoderm^8^,^9^. In humans, EVX1 is thought to function in a regulatory network with GOOSECOID (GSC) and BRACHYURY (T) to control anterior-posterior cell fates^10^. Recent evidence also points to it being a direct target of the WNT signalling pathway, as β-catenin binds to the *EVX1* promoter in differentiating hESCs^11^. Other related members of ‘non-clustered’ Hox gene families play master regulatory roles in A-P and dorsal-ventral (D-V) patterning in *Xenopus laevis*, zebrafish and mammals. For example, *Mixl1* is essential for posterior fate specification in mice^12^, and many Mix/Bix family members are required for ventral specification in response to BMPs in frogs^13^,^14^,^15^,^16^,^17^. *Mezzo*^18^, *dharma*/*bozozok*^19^ and *Mtx1*^20^ are required for formation or patterning of mesendoerm in zebrafish, and goosecoid or related anteriorly-expressed genes are required for anterior specification in most vertebrate species^21^.

Initially, EVX1 was thought to be critical for murine development, as EVX1^−/−^ embryos were reported as failing to implant in the uterine wall^22^. However, other groups subsequently demonstrated that EVX1 is not required for viable embryonic development and suggested the initial EVX1^−/−^ phenotype was due to disruption of another closely linked gene^23^ (Gail Martin, personal communication). In fact, EVX1^−/−^ mice display no overt signs of gastrulation defects after birth, other than minor tail kinks in some offspring and genetic backgrounds (Gail Martin, personal communication). Therefore, the importance of EVX1 in the regulation of gastrulation remains controversial.

*Evx1* is expressed from a complex locus, which also expresses a long non-coding RNA (lncRNA), known as *Evx1as*^5^. There are other well studied lncRNAs associated with the HoxA cluster, including *Haunt/Halr1* which is located ~40 kb upstream of *Hoxa1*^24^, and *HOTTIP* which is immediately downstream of *Hoxa13*, the most 3′ member of the cluster^25^ (Fig. 1). Both have complex functions in global regulation or fine tuning of expression of the HoxA cluster. LncRNAs are an emerging class of regulatory molecules which have been implicated in a number of developmental processes^26^,^27^,^28^,^29^. They have been proposed to function via regulation of transcription, splicing, and chromatin dynamics. Antisense/bidirectional transcription of protein coding genes and lncRNAs is a common feature in the mammalian genome, in particular at highly expressed and developmentally regulated genes^30^. We have previously reported *Evx1* and *Evx1as* are dynamically and concomitantly expressed during embryoid body (EB) differentiation^5^, a commonly used model of early *in vivo* embryonic development and the function of lncRNAs^24^,^31^. *Evx1as* is abundant and has stability similar to *Evx1* transcripts, suggesting function^5^.

We sought to dissect the *Evx1/Evx1as* locus in order to elucidate the potential role for EVX1 and/or *Evx1as* during gastrulation, and to gain insights into the broader functional significance of antisense/bidirectional lncRNAs. To investigate the function of EVX1, we generated bi-allelic small frameshift deletions in the homeodomain-encoding region using CRISPR/Cas9. We generated stable murine ES cell clones and performed RNAseq at day 4 of embryoid body (EB) differentiation in direct comparison with parental non-edited ES cells. We found that disruption of EVX1 results in upregulation of anterior visceral endoderm (AVE) and definitive endoderm genes including *Cer1* and *Sox17*, at the expense of posterior genes, such as *Mixl1* and *Kdr* (Flk-1). We also show EVX1 is likely to function as a downstream effector of BMP4 and WNT signalling pathways, to regulate posterior tissue patterning.

To test whether *Evx1as* has a function independent of EVX1, we generated a suite of CRISPR-Cas9 mediated deletions using a similar approach to that recently reported for *Haunt*^24^. With all of these DNA manipulations, we were not able to disrupt expression of *Evx1as* without also disrupting expression of *Evx1*. We performed RNAseq to convincingly show loss of EVX1 and *Evx1as* produced identical aberrations in A-P gene expression patterns to those which we observed in the EVX1 loss-of-function cell lines. Together, our results strongly suggest there is no independent function for *Evx1as* beyond that of EVX1. However, we cannot rule out a function for *Evx1as* in the regulation of *Evx1 in cis*.

## Results

### Organization, conservation and expression of the *Evx1/Evx1as* locus

**Figure 1:**
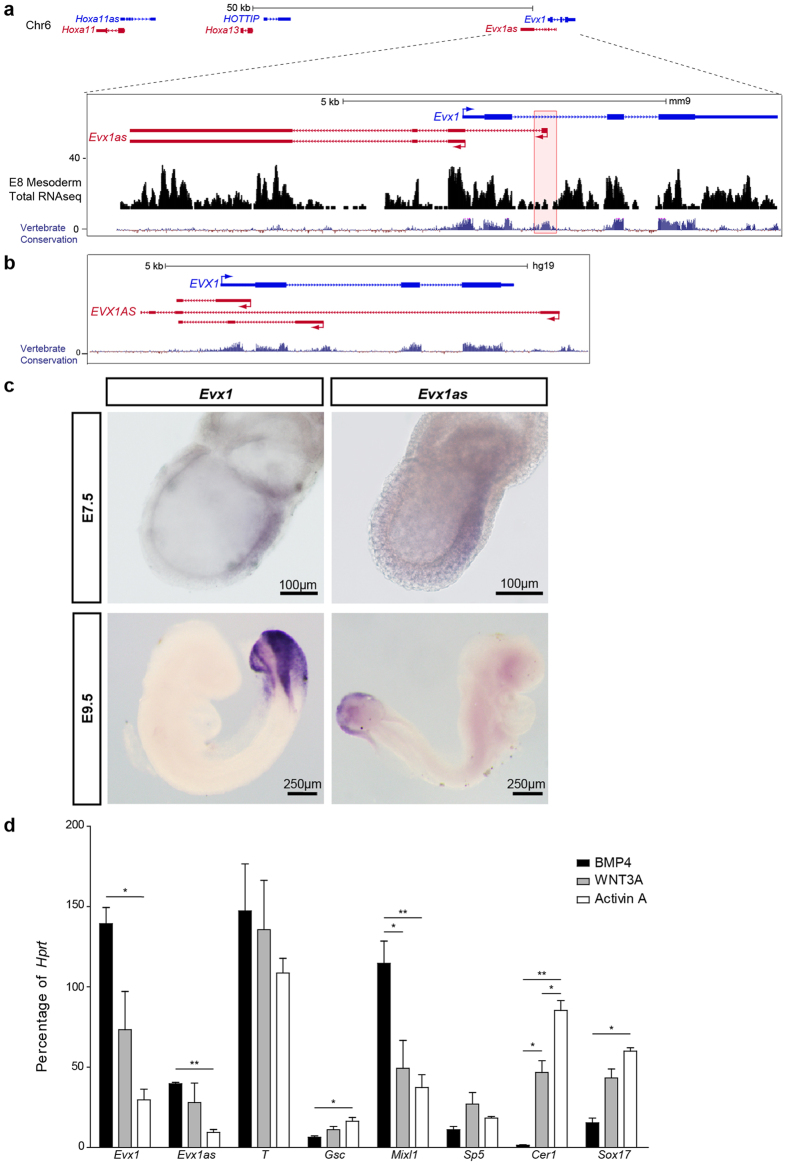
*Evx1* and *Evx1as* are co-expressed in the primitive streak during gastrulation. (**a**) Schematic of the *Evx1/Evx1as* locus, and its proximity to the HoxA cluster modified from the UCSC genome browser. Wiggle track of Total RNAseq from E8 mouse Pre-Somatic Mesoderm (PSM) and Vertebrate Conservation tracks are also shown. Conservation of the *Evx1as* P1 region is boxed in red. (**b**) UCSC browser shot of the human *EVX1* locus and the syntenic *EVX1AS* transcripts. (**c**) WISH of E7.5 and E9.5 mouse embryos using probes against *Evx1* and *Evx1as*. Purple indicates the presence of the transcript. (**d**) Expression profiling of mESCs differentiated to day 4 EBs under serum-free conditions with the addition of BMP4 (10 ng/ml), WNT3A (20 ng/ml) or Activin A (10 ng/ml). Three replicates were performed for each condition. *Indicates p-value < 0.05, **indicates p-value < 0.01. All error bars indicate SEM.

The *Evx1* locus is located 50 kb downstream of the HoxA gene cluster on chromosome 6 (Fig. 1a). *Evx1* and *Evx1as* are developmentally regulated, displaying peak and concordant expression during gastrulation^5^. They are both also highly expressed *in vivo* in the pre-somitic mesoderm (PSM)^32^. Whole mount *in situ* hybridization (WISH) of E7.5 and E9.5 embryos demonstrates the *Evx1* and *Evx1as* are co-expressed in the primitive streak during gastrulation (Fig. 1c). At E7.5, both *Evx1* and *Evx1as* are expressed at the posterior-proximal side of the embryo, which is the location of the primitive streak. At E9.5, both transcripts localize to the tail bud, which contains the embryological remnants of the primitive streak. Thus, *Evx1* and *Evx1as* are co-expressed during gastrulation.

Like many lncRNA-coding gene pairs^30^, *Evx1* and *Evx1as* are expressed from opposite DNA strands in a sense-antisense configuration (Fig. 1a). Interestingly, there are two other lncRNAs within the distal end of the HoxA cluster, *Hox11as* and *HOTTIP*^25^, which are expressed in an antisense direction with respect to *Hox11* and *Hox13* coding genes, respectively (Fig. 1a). *Evx1as* is expressed as at least two different isoforms according to EST and depostied cDNA data; the second exon overlaps with the start of *Evx1* by ~70 bp and the first exon resides in the first intron of *Evx1* (Fig. 1a). We confirmed only 1% of the *Evx1as* transcript contains the first exon in D4 EBs (Supplementary Figure 1A). EVX1 is highly conserved throughout the protein coding sequence, whereas *Evx1as* displays notable conservation at the promoter (Fig. 1a), like many lncRNAs^33^. The human *EVX1-EVX1AS* locus is syntenic with the murine locus. *EVX1AS* has two conserved promoters (intronic or bidirectional), but is also expressed from one additional promoter, located 3′ of EVX1 (Fig. 1b).

### EVX1 regulates anterior-posterior patterning during gastrulation

In order to study the function of EVX1 and *Evx1as*, we used EB differentiation as a model for early *in vivo* mouse embryonic development. Day 4 EBs grown in serum or serum free media (SFM) with recombinant BMP4 display a very similar transcriptional program to embryos undergoing gastrulation^7^. *Evx1* and *Evx1as* were most highly expressed when differentiated under BMP4 and WNT3A conditions (Fig. 1d), which suggests that they might be either downstream targets of the BMP4 and WNT3A signalling pathways or that BMP4 and WNT3A enhance the generation of posterior mesoderm cell types, wherein the *Evx1* locus is expressed. *Evx1* and *Evx1as* were expressed at a much lower level in EBs grown in SFM with Activin A, which is an anterior/dorsal mesoderm inducing factor^34^. Our results are consistent with expression of human EVX1 in these same growth factors^10^. *Evx1* and *Evx1as* display a similar expression pattern to *Mixl1* (Fig. 1d), a related homeodomain transcription factor which is also expressed in nascent posterior mesoderm^12^. Expression of pan-mesoderm genes such as *Brachyury* (T) and *Sp5* was similar in BMP4, WNT3A and Activin A, while expression of anterior markers *Cer1* and *Sox17* was significantly higher in WNT3A and Activin A (Fig. 1d).

**Figure 2 f2:**
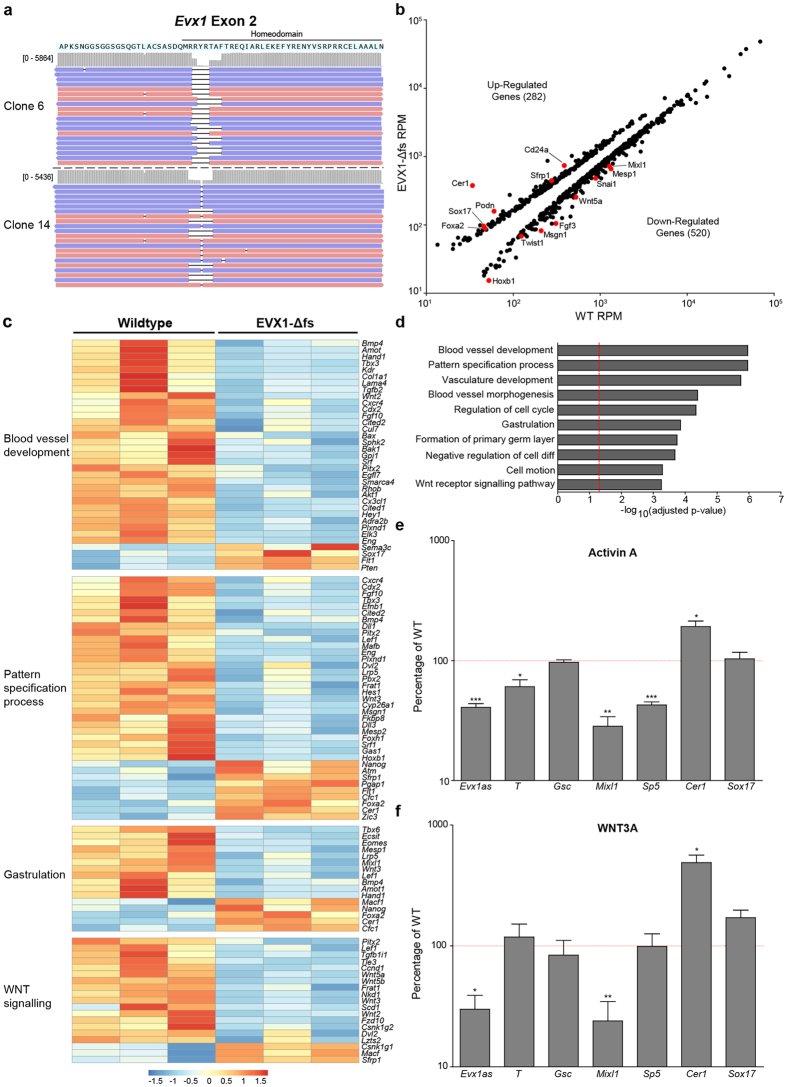
EVX1 is required to regulate anterior-posterior patterning during gastrulation. (**a**) Visualization of CRISPR-induced mutations in two independent mESC clones (clone 6 and 14) using Integrated Genome Viewer (IGV). The peptide sequence of EVX1 is shown. Red and blue indicate different read strands. (**b**) Scatterplot of average counts of DEGs from mRNAseq comparing 3 replicates of WT and EVX1-Δfs Day 4 EBs. Known markers and regulators of tissue specification are shown in red. RPM = Reads per million reads.(**c**) Heatmap of counts for differentially expressed genes corresponding to particular Gene Ontology (GO) terms in (**d**). Each category is hierarchically clustered. Each value is normalized to its mean expression across all samples. Red indicates higher than average expression, blue indicates lower than average expression. (**d**) GO analysis (DAVID) of DEGs shows the biological processes disrupted in the EVX1-Δfs D4 EBs. Red line indicates an adjusted p-value of 0.05. (**e,f**) Expression profiling of EVX1-Δfs in SFM supplemented with (**e**) Activin A (10 ng/ul) or (**f**) WNT3A (20 ng/ul). Expression of each gene in each sample was first normalized to *Hprt* then normalized to WT expression. 4 biological replicates were performed. *Indicates a p-value < 0.05, **indicates a p-value < 0.01, ***indicates a p-value < 0.001 when compared to WT. All error bars indicate SEM.

**Figure 3:**
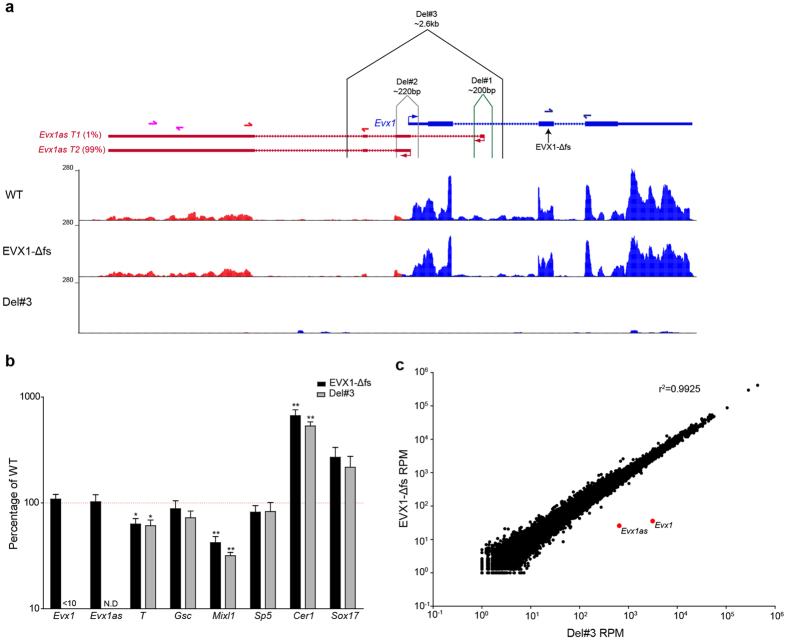
*Evx1as* does not have a function independent of EVX1. (**a**) Schematic of the strategy for dissecting the *Evx1*/*Evx1as* locus modified from USCS Genome Browser. The three different deletions performed are shown on the diagram. Wiggle tracks of WT, EVX1-Δfs and Del#3 mRNAseq from D4 EBs are shown. Corresponding qPCR primers pairs are shown in the same colour. The location of the *Evx1* deletion is indicated by the arrow. Del = deletion, T1 = transcript 1, T2 = transcript 2. (**b**) Expression profiling of EVX1-Δfs and Del#3. Expression of each gene in each sample was first normalized to *Hprt* then normalized to WT expression. 4 biological replicates were performed. Error bars show SEM. *Indicates a p-value < 0.05, **indicates a p-value < 0.01, when compared to WT. No significant differences were found when comparing EVX1-Δfs and Del#3. N.D = not detected. <10 = less than 10% of WT. (**c**) Scatter plot of average mRNAseq counts from three replicates comparing EVX1-Δfs and Del #3 D4 EBs. *Evx1* and *Evx1as* are shown in red. A value of 1 was added to all RPM values to improve visualization. R^2^ was obtained from Pearson Correlation. RPM = Reads per million reads.

We generated EVX1 ‘knockout’ cell lines using CRISPR-Cas9 gene micro-editing of the homeodomain coding region in exon 2, thus leaving *Evx1as* unperturbed [Fig f2](Fig. 3a). Two independent compound heterozygous *Evx1* gene edited clones were selected based on our previously described NGS screening strategy^35^. Both clones have small frameshift mutations in both alleles (Fig. 2a), which are predicted to result in a premature stop codons within 20 bps of the deletion site. Thus, no functional DNA-binding domain of EVX1 could be generated by either of these clones. We refer to these CRISPR-edited lines as ‘EVX1-Δfs’ from here on. EVX1-Δfs lines and W9.5 controls were differentiated in serum to D4 under suspension culture conditions to identify a potential regulatory role for EVX1 during gastrulation. EVX1-Δfs D4 EBs appeared phenotypically normal, and indistinguishable from the wildtype controls (Supplementary Figure 1B). However, qPCR analysis revealed that both EVX1-Δfs clones display differences in expression of key anterior and posterior patterning markers, including *Mixl1* and *Cer1* (Supplementary Figure 1C). There were no significant differences between the two clones, therefore only one was used for subsequent analyses (Clone 14) (Supplementary Figure 1C).

To determine whether EVX1 has a global role in anterior-posterior patterning, mRNA-seq was performed on three biological replicates of W9.5 and EVX1-Δfs EBs differentiated for 4 days in serum without LIF (see Methods). Using DESeq2, we identified 802 differentially expressed genes (DEGs). In the absence of functional EVX1, 282 genes were upregulated and 520 genes were downregulated (Fig. 2b). The upregulated genes included many which are preferentially expressed in anterior visceral endoderm and/or anterior mesendoderm (e.g. *Sox17, Cer1*, and *Foxa2*). Many of the down regulated genes are normally expressed in posterior mesoderm (e.g. *Mixl1, Mesp1, Wnt5a* and *Fgf3*) (Fig. 2b). This suggests there is an imbalance in cellular composition of EVX1-Δfs EBs with loss of posterior cell types and relative expansion of anterior cell types (both mesoderm and endoderm).

Using Gene Ontology (GO) we found the most highly significantly enriched cell biological process terms among the DEGs were: blood vessel development (35) and morphogenesis (28), pattern specification (38), regulation of the cell cycle (29), gastrulation (16) or formation of primary germ layer (12), cell differentiation (25), cell motion (37) and WNT signalling (20) (Fig. 2d). We undertook hierarchical clustering of the genes within each of the GO Ontology categories (Fig. 2c). Each category contains genes down-regulated (blue) or up-regulated (red) in EVX1-Δfs cells. Within the blood vessel development, patterning and gastrulation categories, there is down-regulation of many ligands, receptors, signalling molecules and transcription factors which are implicated in posterior patterning of mesoderm. These include *Bmp4, Wnt2, Fgf10, Kdr (Flk1*), *Cxcr4, Cited1, Cited2, Cdx2, Hand1, Mixl1, Hes1, Tbx3, Tbx6, Mesp1, Eomes, Snai1* and others. There is also upregulation of a smaller set of key patterning genes which are normally expressed in the AVE, anterior mesendoderm or epiblast. These include *Sox17, Nanog, Atm, Zic3*, and *Foxa2*. We also found many genes in the WNT signalling pathway are downregulated in EVX1-Δfs EBs, including *Wnt3, Wnt2, Wnt5a, Wnt5b, Lef1, Pitx2, Dvl2, Lrp5* and others. On the other hand, WNT pathway antagonists such as *Cer1, Cfc1/cripto* and *Sfrp1* are upregulated. Interestingly, *Evx1* is downregulated in WNT3A KO embryos^36^, demonstrating that there is a mutual dependence (direct or indirect) between WNT signalling and EVX1. There were no significant changes in expression of any members of the HoxA cluster under these differentiation conditions. Full lists of differentially expressed genes are provided in Supplementary Table 2.

Previously EVX1 has been shown to directly repress GSC in human ESCs under Activin A growth conditions^10^. *Gsc* was not upregulated in either EVX1-Δfs clone by qPCR or RNAseq under serum differentiation conditions (Supplemental Table 2, Supplementary Figure 1C). To ensure that the lack of *Gsc* upregulation was not due to differences in the differentiation conditions used, we differentiated EVX1-Δfs and W9.5 EBs in SFM with Activin A. We did not find significant upregulation of *Gsc* (Fig. 2e). Importantly, the A-P gene expression defect in EVX1-Δfs persisted in Activin A. Defective expression of *Mixl1* and *T* remained, and additional significant reduction in expression of the pan mesoderm marker *Sp5* was also present in EVX1-Δfs EBs (Fig. 2e). The upregulation of AVE and endoderm markers *Cer1* and *Sox17* was less dramatic in Activin A, presumably because selective expansion of AVE and anterior tissues dilutes the differences in anterior gene expression between wildtype and EVX1-Δfs EBs (Fig. 2f).

From this RNAseq data, it was unclear whether EVX1 exerts its A-P patterning function by directly regulating BMP4 and/or WNT signalling pathways or whether changes in *Bmp4/Wnt* expression are an indirect result of changes in cellular composition within the EVX1-Δfs EBs. BMP4 and WNT direct EB differentiation towards posterior cell types^37^, so the aberrant transcriptome in EVX1-Δfs EBs may be primarily due to downregulation of these factors. To address this possibility, we attempted to rescue EVX1-Δfs EBs by culture in SFM with exogenous recombinant WNT3A (20 ng/ml) or BMP4 (10 ng/ml). Under WNT3A conditions, we observed no rescue of *Mixl1* gene expression; i.e. it remained significantly down regulated (Fig. 2f). Likewise there was no reversal of the up-regulation of *Cer1*, suggesting the A-P patterning defect was not rescued by WNT3A (Fig. 2f). Similar results were found for EBs grown in BMP4 (Supplementary Figure 1D). Thus, downregulation of *Bmp4* and *Wnt* genes (*Wnt3, Wnt5a* and *Wnt5b*) is likely to be an indirect result of changes in cellular composition of EVX1-Δfs EBs and not due to direct regulation of these factors by EVX1. This data also shows EVX1 is required for appropriate transcriptional responses and differentiation downstream of BMP4 and WNT signalling pathways. This function appears to be conserved in humans, as β-catenin, the major effector of WNT signalling, has been shown to bind to regulatory regions in the *EVX1* promoter in differentiated hESCs^11^. Together, these results confirm EVX1 plays a key role in A-P patterning of nascent mesoderm and endoderm and is essential for regulating the posteriorizing effects of WNTs and BMP4.

### CRISPR/Cas9 mediated removal of the *Evx1/Evx1as* locus results in an identical D-V pattering defect as EVX1-Δfs

Bidirectional/antisense transcription from highly expressed, developmentally regulated genes is a common phenomenon in the mammalian genome^30^. However the functional significance and possible mechanisms of action of such antisense lncRNAs remain topics of debate^38^,^39^. The *Evx1* locus contains a stable, highly expressed antisense lncRNA associated with *Evx1*, so it is an ideal locus to study. Visual inspection of RNAseq data from D4 EBs suggests that the majority of the *Evx1as* transcript is expressed as *Evx1as* T2 (Fig. 3a). We further validated this by qPCR using primers to distinguish between the different isoforms of *Evx1as*. Only 1% of the total transcript derived from the overlapping intronic promoter (Supplementary Figure 1A).

We initially used RNAi, delivered via shRNA containing lentiviral vectors^40^ to attempt stable knockdown of *Evx1as* without disturbing the genomic locus (Supplementary Figure 2A) (see Methods). Such an approach has been successful for some lncRNAs but challenging for others^41^,^42^. In some cases RNAi has obtained different and more severe phenotypes than constitutive deletions, which may be due to off target or toxicity effects in some cases or due to compensatory events in others^43^,^44^. We generated stable clones targeting *Evx1as* using three independent shRNAs (Supplementary Figure 2A). Unfortunately, none of the clones from any of the three siRNAs displayed knockdown of *Evx1as* to <50% of WT levels, so we could not make concrete conclusions about the potential function of *Evx1as* from these experiments. There were no significant changes in expression of key mesoderm genes such as *Brachyury, Mixl1*, or *Evx1* itself (data not shown).

Genetic deletion or mutation remains the gold standard to demonstrate the requirement of a gene’s function^43^. We therefore performed 3 different CRISPR-Cas9 mediated manipulations of the *Evx1/Evx1as* locus (Fig. 3a). The first manipulation ‘Del#1’ removed the entire first intronic promoter of *Evx1as*, which is specific to isoform *Evx1as* T1 (1% of total transcript) and conserved (Fig. 1a). Removal of this promoter, which generates a small proportion of *Evx1as* RNA, did not affect expression of *Evx1as* or *Evx1* (data not shown). ‘Del#2’ removed the shared promoter region between *Evx1* and *Evx1as*. This deletion removed 220 bps centred around the beginning of *Evx1* and *Evx1as* transcription, based on ESTs and RNAseq data. This deletion resulted in a dramatic loss of *Evx1* reducing the transcript to ~16% of WT levels (Supplementary Figure 2B). However this deletion only decreased *Evx1as* to ~46% of WT levels. This result may be explained by a more malleable transcriptional start site and/or weaker constraints on transcriptional regulation at the lncRNA promoter. As expected, ‘Del#2’ displayed reduction of *Mixl1* and significant upregulation of *Cer1*, supporting the results from the EVX1-Δfs RNAseq data (Supplemental Figure 2B).

Since *Evx1as* was still expressed at 46% of WT in ‘Del#2’, we could not draw any definitive conclusions about its potential function. Therefore, we generated a large ~2.6 kb deletion (‘Del#3’) that removed the promoters from both versions of the transcript (to prevent the possibility of isoform switching between the two alternative promoters), as well as the second/third exon (Fig. 3a). Successful deletion was validated by PCR and later by RNAseq (Fig. 3a, Supplementary Figure 2C). This deletion resulted in almost complete ablation of *Evx1* by qRT-PCR (Fig. 3b). Since the *Evx1as* qPCR primers sit inside the deletion site (Fig. 3a), primers were designed for the last exon of the transcript to detect any residual expression. Approximately 15% of WT levels of expression of the last exon of *Evx1as* remained in the ‘Del#3’ D4 EBs (Supplementary Figure 2D).

To determine whether *Evx1as* has any function independent of EVX1, we performed a direct comparison between the transcriptome of EVX1-Δfs and the *Evx1as*/*Evx1* double KO (‘Del#3’). We reasoned that if *Evx1as* has an independent function from EVX1, then differences should be observed between the two transcriptomes, regardless of whether it functions in transcriptional or post-transcriptional regulation. Transcriptional differences, if present, could be either due to direct transcriptional regulation by *Evx1as* or an indirect consequence of *Evx1as* regulating post-transcriptional pathways.

No significant differences were observed by qPCR between the two lines for a number of gastrulation and A-P patterning genes (Fig. 3b). Both the EVX1-Δfs and *Evx1/Evx1as* Δ2.6 kb (‘Del#3’) EBs displayed significantly different expression of *Mixl1, T* and *Cer1* relative to WT EBs (Fig. 3b). To determine whether there was any *trans* function for *Evx1as* RNA independent of the function of EVX1, we searched for differences genome-wide by mRNAseq. Remarkably, differential gene expression analysis identified only 3 DEGs, two of which were *Evx1* and *Evx1as*, themselves. In the *Evx1/Evx1as* double KO (‘Del#3’), *Evx1* and *Evx1as* are dramatically reduced to 1% and 4% of EVX1-Δfs (in which *Evx1* and *Evx1as* are expressed the same as WT under serum conditions). The other DEG (*Grb10*) has no reported role during gastrulation and is unlikely to be of any biological significance. The lack of differences is reflected in the very high correlation observed between the two samples (R^2^ = 0.9925, Pearson) (Fig. 3c). We therefore conclude that *Evx1as* does not have function independent of EVX1, and therefore is unlikely to function *in trans*. This does not rule out a potential function for *Evx1as* in the regulation of *Evx1 in cis* (see Discussion).

## Discussion

Anterior-posterior patterning determines cell fates and tissue specification during gastrulation. Although, EVX1^−/−^ mice are viable and display no dramatic gastrulation phenotype after birth, we show EVX1 is an important downstream effector of BMP4 and WNT signalling in EBs, and regulates A-P patterning. Genes which are normally highly expressed on the posterior side of the embryo are markedly reduced in EVX1-Δfs EBs. Thus, many genes associated with blood, blood vessel and cardiac outcomes (e.g. *Kdr (Flk1), Hand1, Cited2, Tbx3*)^45^,^46^,^47^,^48^,^49^ are markedly downregulated in the absence of EVX1. On the other hand, genes expressed throughout the mesoderm such as *Brachyury (T*) and *Sp5*^50^ are only mildly affected. Genes expressed on the anterior-dorsal side of the embryo such as *Cer1* and *Sox17*^51^ are increased. This is consistent with the functions of EVX1 in *Xenopus laevis (xhox3*), zebrafish (*eve1*) and humans (EVX1); i.e. they all play essential roles in posteriorization of nascent mesoderm^8^,^9^,^10^.

Members of the homeobox gene superfamily can act as either transcriptional activators and/or repressors depending on cofactors or context. EVX1 may function as a transcriptional activator of posterior mesoderm genes, such as *Mixl1*, and/or as a transcriptional repressor of anterior/dorsal genes such as *Sox17* and *Cer1*. Thus, it could function to maintain the integrity of the posterior region of the embryo by preventing ectopic expression of anterior genes. Indeed, such a role has been proposed for human EVX1^10^. Both scenarios are possible and not mutually exclusive. For example, EVX1 might co-operate with transcriptional activators or repressors, or recruit co-activators and/or co-repressors in different contexts. ChIPseq for EVX1 would be able to distinguish these possibilities; however, no specific mouse EVX1 antibodies are available and we found human antibodies did not work in the mouse.

As is the case for EVX1, disruption of some other key regulators of gastrulation does not always result in strong gastrulation phenotypes in the intact mouse. For example, the GSC, SP5 and CER1 knockout mice display no overt signs of gastrulation defects, despite each of these genes having well established roles in regulating patterning in ES cells or model organisms^36^,^52^,^53^,^54^,^55^. The lack of an overt phenotype may be explained by genetic redundancy and/or compensation by other genes and pathways. This is the case for *Cer1*, which has an overlapping expression profile and redundant function with *Lefty1*^56^. Unlike either of the single knockout mice, the CER1^−/−^/LEFTY1^−/−^ mouse has an expanded primitive streak or multiple primitive streaks^56^. Whether redundancy explains the lack of phenotype in the EVX1^−/−^ mice is unclear. *Evx1* has a paralog, *Evx2*, which is thought to have arisen from vertebrate specific duplication of *Evx1*^57^. It is similar to *Evx1* (82.5% at the nucleotide level), and only differs at one amino acid in the homeodomain^58^. However, *Evx2* is not expressed during gastrulation *in vivo*^57^ or in EBs, so it is unlikely to act redundantly with *Evx1*.

Compensation within key signalling pathways has been shown to ‘buffer’ mis-regulation of some genes during development and dampen phenotypic consequences of gene knockouts^59^,^60^,^61^,^62^. Since BMP, WNT, TGFβ and FGF signalling pathways act on a large number of genes, it is possible that changes in expression or activity of components of these pathways could compensate for the loss of EVX1 in the context of an entire embryo^1^,^59^,^61^. This does not appear to occur in EVX1-Δfs EBs, since BMP4 and WNT3A cannot rescue the posterior mesoderm gene expression program. Perhaps only in the context of an intact embryo, can compensation be fully active. Alternatively, compensation may occur at the level of alternated expression of transcription factors (such as *Mixl1*) *in vivo*, but not in EBs. In short, it remains unclear why EVX1^−/−^ mice display minimal defects, despite a clear function for EVX1 in regulating A-P patterning in EBs.

LncRNAs are an important new class of genetic material with a variety of biochemical activities. Some have proven key regulatory roles in development and differentiation. The abundance of sense-antisense coding-lncRNA gene pairs, such as *Evx1*/*Evx1as*, is widely appreciated but the functional significance of the associated lncRNA transcript remains mostly untested. This is partly because functional studies have focused primarily on intergenic lncRNAs to avoid disrupting overlapping coding transcripts^63^. Certainly, some lncRNAs have well established functions *in trans*^28^,^63^. However, despite being stable, spliced, abundant and developmentally regulated, *Evx1as* does not function to regulate gene expression independently of EVX1; it is therefore unlikely to function *in trans*. This does not rule out a possible *cis* function for *Evx1as* in the regulation of *Evx1*. Indeed, other examples of *cis* acting lncRNAs have been reported^5^,^25^,^64^,^65^. Further coding-lncRNA pairs need be carefully dissected using precise genetic tools to develop general principles which accurately describe their biological functions. We suggest that it is likely that some lncRNAs may function *in trans* to exert broad effects on gene expression, some function in *cis* to regulate their coding partner, and some may simply be non-functional bi-products of intense transcriptional activity at the partner coding gene’s promoter or enhancer.

## Methods

### ESC Culture and embryoid body differentiation

W9.5 mESCs were maintained on gelatanized plates in mESC media with 1000 U/ml LIF at 37 °C, 5% CO_2_, as described previously^7^. To generate EBs, mESCs were plated at a density of 2 × 10^4^ cells/ml on STAR^TM^ low adherence plates and allowed to differentiate for 4 days in suspension. Serum free differentiation was conducted in ESGRO complete basal media (SF002, Millipore) as described previously^66^. Serum-free media was supplemented with recombinant BMP4 at 10 ng/ml, recombinant Activin A at 10 ng/ml and recombinant WNT3A at 20 ng/ml. BMP4, Activin A and WNT3A were purchased from R and D Biosystems. Differentiations performed for side by side comparisons between conditions were set up on the same day to minimize technical and biological variation. At least 3 differentiations (biological replicates) were performed for each comparison with each replicate set up on a different day.

### RNA extraction and qRT-PCR

RNA was extracted from cells harvested at D4 using Trizol^TM^ (Thermo-Fischer) according to manufacturer’s instructions. cDNA was synthesized using Superscript III^TM^ (Thermo-Fischer) according to manufacturer’s instructions using random hexamer primers. qRT-PCR was performed using SYBR-Green (Thermo-Fischer) on the AB7900 (Applied Biosystems). Delta Ct was calculated by comparing to *Hprt* as a house keeping gene. Delta Delta Ct was calculated by normalizing each condition to WT expression levels. Statistical significance was determined by t-tests. Each condition was tested for significantly different expression to WT. At least 3 biological replicates were performed for each condition in each experiment. For testing the relative abundance of each *Evx1as* isoform, standard curves were performed with known quantities of DNA to calculate primer efficiencies. These primers efficiencies were taken into account in the Delta Ct calculations. All graphs and statistical tests were performed in Prism 5 (GraphPad). Primer sequences are available in Supplementary Table 1.

### CRISPR-Cas9 clone generation and screening

All sgRNAs were designed using the CRISPR design tool (crispr.mit.edu) and cloned into the pSpCas9(BB)-2A-GFP plasmid (#48138, Addgene) as described previously^67^. pSpCas9(BB)-2A-GFP (PX458) was a gift from Feng Zhang. For generating the EVX1-Δfs mESCs, a single guide was targeted to the second exon of *Evx1* in the homeodomain region. mESCs were transfected with the pCas9-GFP plasmid also expressing the sgRNA targeting *Evx1*. Sorting and screening was conducted as described previously, with minor modifications^35^. Specifically, the top 5% of GFP + cells were sorted into a 10 cm^2^ dish and allowed to grow for two days before picking individual clones. This was done to prevent high numbers of mixed clones, which results from retention of the plasmid through cell division. Large deletions (‘Del#1′, ’Del#2′ and ‘Del#3′) were generated using the dual sgRNA strategy^67^. mESCs were transfected with two plasmids, each containing pCas9-GFP and the appropriate sgRNA. The top 5% of GFP + cells were sorted into a 10 cm^2^ dish and allowed to grow for two days before picking individual clones. Individual clones were transferred to a 96 well plate and allowed to grow until confluent. DNA was extracted and PCR was performed using primers outside and inside the deleted region to identify clones containing the intended deletions. Clones with the appropriate deletion were expanded and used for subsequent analyses. sgRNA and screening primer sequences are available in Supplementary Table 1.

### Whole mount *In Situ* Hybridization (WISH)

WISH was conducted as described previously, with minor modifications to reduce Proteinase K digestion of E7.5 embryos^5^. Primers used to amplify and clone probes for *Evx1* and *Evx1as* are available in Supplementary Table 1.

### RNA Sequencing and data analysis

Total RNA was analysed on the Bioanalyser (Agilent) to confirm high RNA quality (Rinn value > 8). mRNA selection of total RNA was performed using Ambion Dynabeads^©^ mRNA DIRECT^TM^ Micro Purification Kit (#61021, Life Technologies). Barcoded RNAseq libraries were generated from the mRNA using the Ion Total RNAseq Kit v3 (#4475936) and Ion Xpress^TM^ RNAseq Barcode 1–16 Kit (#4475485, Life Technologies). RNAseq libraries were sequenced on the Ion Proton sequencing instrument using the Ion P1 Hi-Q Sequencing 200 Kit with the Ion P1 v2 chip. Each sample was sequenced to a depth of least 15 million reads. RNAseq data was mapped to the Mus Musculus reference genome (mm9) using TopHat2 and then TMAP^68^. Tophat2 is required to map spliced junctions, while TMAP is designed for IonTorrent sequencing data. Transcript counts were obtained by using HTSeq on the mapped data using the standard settings^69^. Differential gene expression analysis was performed on the HTSeq output using DESeq2 using the standard settings^70^. Hierarchically clustered heatmaps were generated in R using pHeatmap (Bioconductor). Scatterplots were generated in Prism 5 (GraphPad). Gene Ontology analysis was performed with all DEGs on DAVID^71^. Raw data is available via the GEO repository (http://www.ncbi.nlm.nih.gov/geo/) under the accession numbers: GSE75735, GSE75737.

### Lentiviral Transduction of shRNAs

We employed the iRNAi v2.1 software to design siRNAs targeting the *Evx1as* gene. We tested for potential off targeting in the genome by performing BLAST searches to exclude candidates which bound to additional genomic sites. We selected three siRNAs for cloning (Supplementary Table 1). We purchased ssDNA oligos designed for annealing to leave 5′ and 3′ overhands for cloning into the *Cla1* and *Mlu1* restriction sites of pLVTHM^40^. We annealed 2 ul of sense and antisense oligos (1 ug/ul) in 1× DNA annealing solution (100 mM potassium acetate, 30 mM Hepes, 2 mM magnesium acetate) in a 50 final volume by heating to 94 °C for 5 mins and slowly cooling at 37  °C. dsDNA was phosphorylated with PNK and then ligated to pLVTHM cut with *Mlu1* and *Cla1* and dephosphorylated with CIAP. Ligations were performed by standard techniques. Plasmid preps were checked for inserts and concatamer exclusion by digestion with *EcoR1* and *Xba1* and Sanger sequencing. To generate lentiviruses, 293T cells were transduced Opti-MEM with Lipofectamine-2000 and shRNA-pLVTHM together with pCMV-dR8.2 and pMD2G-VSV-G, as described^40^. Viral stocks were harvested from media at 48–72 hours, filtered (0.45 μ) and snap frozen. For ES cell transductions, adherent ES cells were cultured for 48 hours with viral supernatant (20% in ES cell media) and polybrene (4 μg/ml). Transduced cells were sorted as eGFP + by FACS and seeded onto MEFs. GFP + clones were then selected for molecular and functional analyses.

## Additional Information

**How to cite this article**: Bell, C. C. *et al*. The *Evx1/Evx1as* gene locus regulates anterior-posterior patterning during gastrulation. *Sci. Rep.* **6**, 26657; doi: 10.1038/srep26657 (2016).

## Supplementary Material

Supplementary Information

Supplementary Table 1

Supplementary Table 2

Supplementary Table 3
