# Supplementary material for: The Evx1/Evx1as gene locus regulates anterior-posterior patterning during gastrulation
Source: Sci Rep. 2016 May 26;6:26657. doi: 10.1038/srep26657 (PMC4880930; doi:10.1038/srep26657)
Supplement: Supplementary Information [file srep26657-s1.pdf]

Supplementary Information:

The *Evx1/Evx1as* gene locus regulates anterior-posterior patterning during gastrulation

Charles C. Bell (1), Paulo P. Amaral (2,8), Anton Kalsbeek (2,5), Graham W. Magor (1), Kevin R. Gillinder (1), Pierre Tangermann (2), Lorena di Lisio (1), Seth W. Cheetham (1,3,8), Franziska Gruhl (1,3,9), Jessica Frith (2,4), Michael R. Tallack (1,2), Ke-Lin Ru (2,4), Joanna Crawford (2), John S. Mattick (5,6), Marcel E. Dinger (3,5,6), and Andrew C. Perkins (1,2,7)

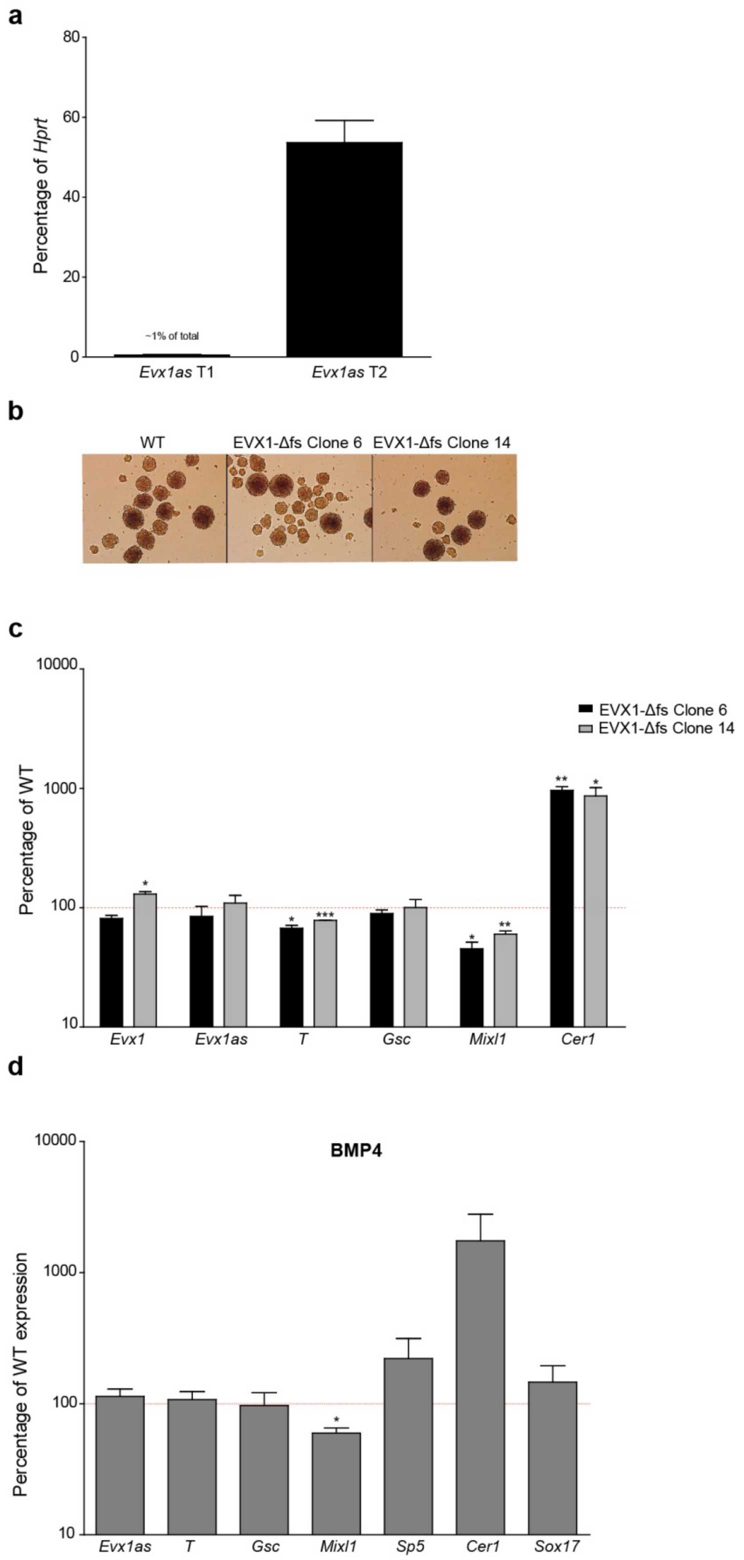
a
80
60
40
20
0
Percentage of Hprt
~1% of total
Evx1as T1
Evx1as T2
b
WT
EVX1-Δfs Clone 6
EVX1-Δfs Clone 14
c
10000
1000
100
10
Percentage of WT
EVX1-Δfs Clone 6
EVX1-Δfs Clone 14
Evx1
Evx1as
T
Gsc
Mixl1
Cer1
d
BMP4
10000
1000
100
10
Percentage of WT expression
Evx1as
T
Gsc
Mixl1
Sp5
Cer1
Sox17

Supplementary Figure 1:

A) qRT-PCR of each isoform of *Evx1as*. Standard curves were performed for each transcript to access primer efficiency. The relative abundance of each transcript is expressed as a percentage of *Hprt*. T1=transcript 1, T2=transcript 2. **B)** Images of D4 EBs at 10x magnification. **C)** Expression profiling of EVX1-Δfs clone 6 and EVX1-Δfs clone 14. Expression of each gene in each sample was first normalized to *Hprt* then normalized to WT expression. 4 biological replicates were performed. No significant differences were found when comparing EVX1-Δfs clone 6 to EVX1-Δfs clone 14. **D)** Expression profiling of EVX1-Δfs in SFM supplemented with BMP4. Expression of each gene in each sample was first normalized to *Hprt* then normalized to WT expression. 4 biological replicates were performed. **C)** and **D)** * indicates a p-value <0.05, ** indicates a p-value <0.01, *** indicates a p-value<0.001 when compared to WT. All error bars show SEM.

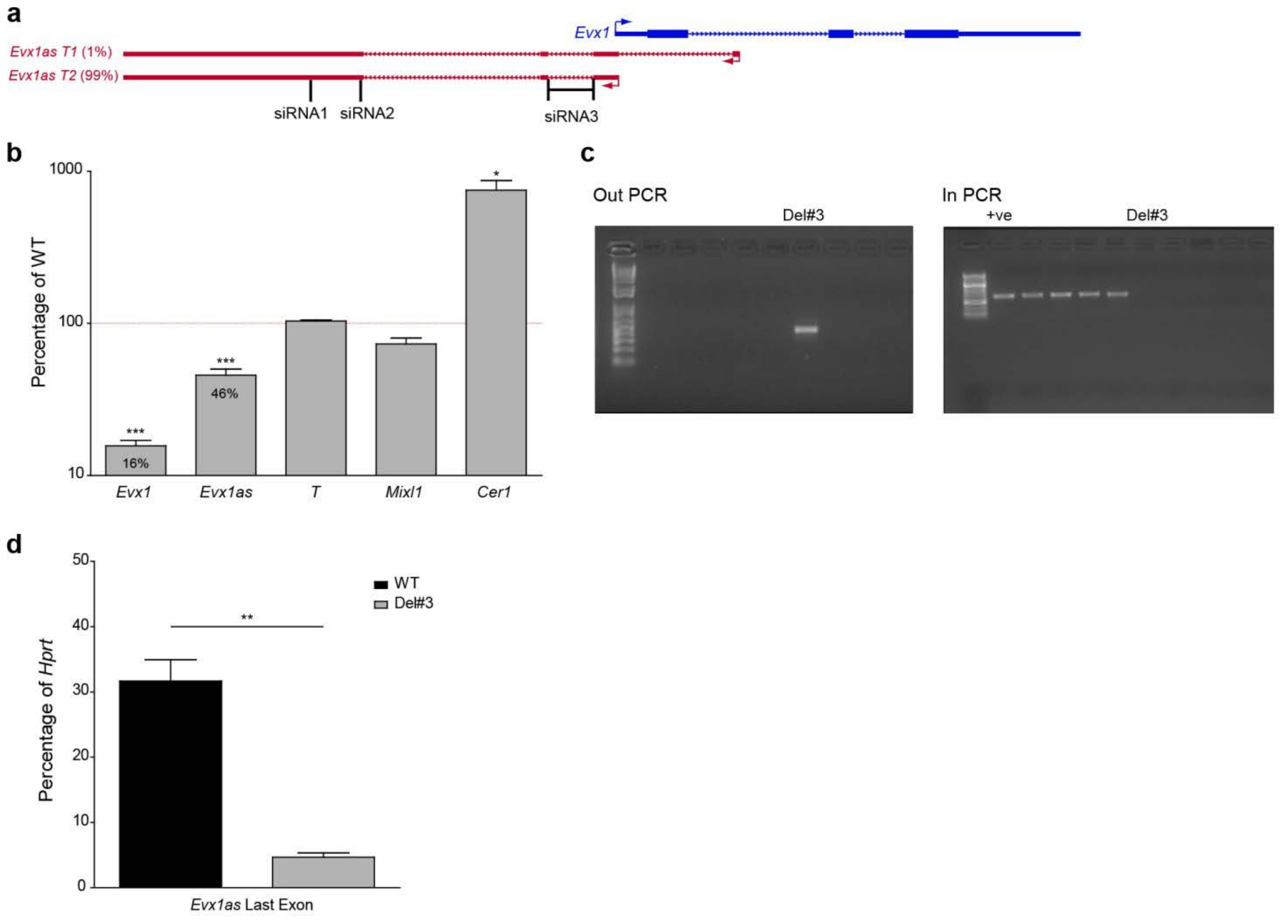

a
Evx1
Evx1as T1 (1%)
Evx1as T2 (99%)
siRNA1
siRNA2
siRNA3
b
1000
100
10
Percentage of WT
***
16%
***
46%
*
Evx1
Evx1as
T
Mixl1
Cer1
c
Out PCR
Del#3
In PCR
+ve
Del#3
d
50
40
30
20
10
0
Percentage of Hprt
WT
Del#3
**
Evx1as Last Exon

Supplementary Figure 2:

A) UCSC browser schematic showing the locations of the three Evx1as siRNAs used. **B)** Expression profiling of Del#2. Expression of each gene in each sample was first normalized to *Hprt* then normalized to WT expression. 3 biological replicates were performed. * indicates a p-value <0.05, ** indicates a p-value <0.01, *** indicates a p-value <0.001 when compared to WT. **C)** Ethidium Bromide gel demonstrating presence of band using Out PCR primers and lack of a band using In PCR primers on Del#3 cells. **D)** qRT-PCR of WT and Del#3 cells using primers designed against the last exon of *Evx1as*. ** indicates a p-value <0.01. All error bars show SEM.
